# Clinicopathological Significance of Neuropilin 1 Expression in Gastric Cancer: A Meta-Analysis

**DOI:** 10.1155/2020/4763492

**Published:** 2020-09-19

**Authors:** Hui Cao, Yan Li, Limin Huang, Banjun Bai, Zhong Xu

**Affiliations:** ^1^Department of Oncology, Guizhou Provincial People's Hospital, Guiyang 550002, China; ^2^Department of Oncology, Chongqing University Cancer Hospital, Chongqing 400030, China; ^3^Department of Gastroenterology, Guizhou Provincial People's Hospital, Guiyang 550002, China; ^4^Department of Infectious Diseases, Guizhou Provincial People's Hospital, Guiyang 550002, China

## Abstract

**Background:**

Neuropilin 1 (NRP1) is involved in tumorigenesis, development, invasion, and metastasis by promoting angiogenesis of tumors. The study is aimed at evaluating the correlation between the expression of NRP1 protein and clinicopathological features of gastric cancer by meta-analysis.

**Methods:**

The published studies were searched in databases including CNKI, Wanfang, Chongqing VIP, Web of Science, and PubMed online. Clinical case studies were included to compare the correlation between NRP1 protein expression and clinicopathological characteristics of gastric cancer. The quality of the included literatures was evaluated by NOS scale. Meta-analysis was performed by Stata software to calculate the odds ratio (OR) and 95% confidence interval (CI).

**Results:**

A total of 12 studies were included in this analysis, involving 1,225 patients with gastric cancer. The analysis indicated that the expression of NRP1 protein in gastric cancer tissues was lower in the group of early stage versus advanced stage (OR = 0.128, 95%CI = 0.059 − 0.277, *P* ≤ 0.001), tumor size less than 5 cm versus more than 5 cm (OR = 0.443, 95%CI = 0.310 − 0.632, *P* ≤ 0.001), TNM stage I-II group versus stage III-IV patients (OR = 0.736, 95%CI = 0.589 − 0.919, *P* = 0.007), well to medium differentiation group versus poor differentiation group (OR = 0.735, 95%CI = 0.632 − 0.854, *P* ≤ 0.001), and nonlymph node metastasis group versus lymph node metastasis group (OR = 0.667, 95%CI = 0.522 − 0.854, *P* ≤ 0.001). The expression of NRP1 protein in gastric cancer was not related to gender, age, and Laurèn's classification.

**Conclusion:**

The expression of NRP1 protein in gastric cancer is closely correlated to clinical stage, tumor size, TNM stage, differentiation, and lymph node metastasis.

## 1. Introduction

Neuropilin-1 (NRP1), a transmembrane nontyrosine-kinase glycoprotein in the neuropilin family, plays an important role in angiogenesis and vasculogenesis [1–3]. Altered expression of NRP1 promotes tumor proliferation, angiogenesis, and metastasis by triggering vascular endothelial growth factor (VEGF) and other pathways [3, 4]. The abnormal expression of NRP1 has been found in various malignant tumors, including chest tumors, abdominal tumors, and nervous system tumors [4]. In gastric cancer (GC), high expression of NRP1 is closely related to the development of tumor progression and associated with poor overall survival [5]. Furthermore, recent report indicated that anti-NRP-1 mAb might be a novel therapeutic approach in the treatment of gastric cancer [6].

For over a decade, a number of studies have examined the expression of NRP1 protein in gastric cancer and analyzed the relationship between the abnormal expression of NRP1 protein and the clinicopathological characteristics of gastric cancer. For instance, Peng [7] reported no difference of NRP1 expression was found between GC patients with and without lymph node metastasis group. Conversely, Yang et al. [8] demonstrated that the positive expression of NRP1 was correlated with lymph node metastasis in GC patients. In addition, Zhang et al. [9] demonstrated no correlation between NRP1 expression and histological differentiation in GC patients. However, Fan et al. [10] concluded that the high expression of NRP1 was associated with differentiation in GC patients. Altogether, the correlation between the expression of NRP1 and the clinicopathological characteristics of GC remains controversial.

To overcome the limited power of individual study with inconsistent results, we used quantitative meta-analysis to evaluate the associations between NRP1 protein expression and the clinicopathological features in gastric cancer.

## 2. Materials and Methods

### 2.1. Literature Search Strategy

Databases including CNKI, Wanfang, Chongqing VIP, Web of Science, and PubMed were searched for relevant studies conducted on human subject until September 2019. There was no restriction for language. Relevant studies were identified in PubMed database by using the following terms: “(Neuropilin∗ or NRP∗) and (gastric OR stomach) and (cancer or carcinoma or neoplasm or tumor)”. Searching strategies in other included databases were adjusted based on these keywords.

### 2.2. Inclusion and Exclusion Criteria

All articles that examined the relationship between the expression of NRP1 and clinicopathological variables of gastric cancer were extracted. The following inclusion criteria were applied for selection: (1) patients with gastric cancer were diagnosed histopathologically, (2) expression of NRP1 protein was measured in patients with gastric cancer by immunohistochemistry, and (3) reported the relationship between the expression of NRP1 and clinicopathological characteristics of patients.

Exclusion criteria were as follows: (1) no sufficient data to calculate the odds ratio (OR) of the NRP1 expression with clinicopathological features; (2) articles of letters, reviews, case reports, and conference abstracts with no original data. If the same research group published data on the same group of patients in more than one journal, the most complete study was selected for the present meta-analysis.

### 2.3. Data Extraction and Quality Assessment

Two investigators (Hui Cao and Zhong Xu) assessed the studies according to inclusion and exclusion criteria. Disagreements were resolved by discussion. Senior reviewers (Banjun Bai) reviewed the final results before next step. The quality of the included studies was evaluated by the Newcastle-Ottawa Scale (NOS) [11, 12]. The following information was recorded from the included studies: first author, publication year, country, number of gastric cancer cases, and NRP1 expression in gastric cancer with clinicopathological features.

### 2.4. Statistical Analysis

Association of NRP1 expression and correlation with clinicopathological features in gastric cancer was estimated by odds ratios (ORs) with 95% confidence intervals (95% CIs), and *P* < 0.05 was considered to indicate statistical significance. Study heterogeneity was determined using the *Q* test and *I*^2^ statistic test (*P* < 0.10 or *I*^2^ > 50% indicated significant heterogeneity). The pooled ORs were calculated by the fixed-effects model when there was no significant heterogeneity. Otherwise, the random-effects model would be adopted. Publication bias was assessed with Begg's funnel plots [13] and Egger's test [14] (*P* < 0.05 was considered representative of statistically significant publication bias). Statistical analyses for the meta-analysis were performed using the software Stata12.0 (Stata Corporation, College Station, Texas).

This study uses the method of our previous publication, and the method description partly reproduces the wording [15].

## 3. Results

### 3.1. Study Characteristics

**Figure 1:**
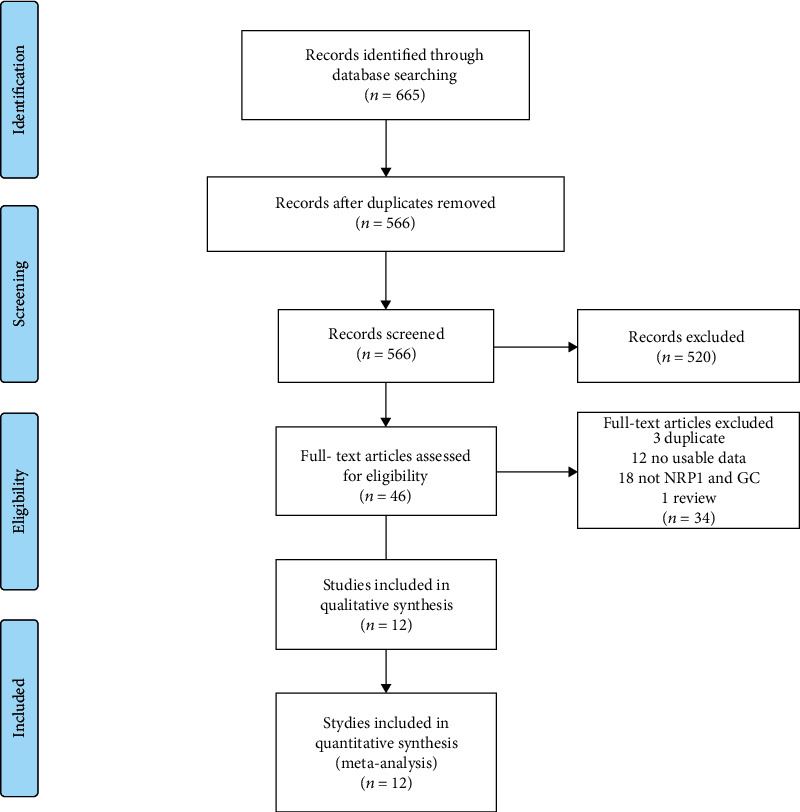
Flow chart of study inclusion and exclusion.

A total of 665 records were identified from online databases for selection. 566 records were included for further screening after removing duplication. The flow chart summarizes the complete literature selection process as shown in Figure 1. Finally, we enrolled 12 eligible studies containing 1,225 gastric cancer patients into our meta-analysis [7–10, 16–23]. Among these studies, 10 were reported in Chinese and 2 in English. All these studies were conducted in China. Characteristics of the included studies in this meta-analysis are summarized in Table 1. The quality scores indicate all of included literatures are high-quality.

### 3.2. Meta-Analysis Results

#### 3.2.1. Gender

All included studies reported the relationship between NRP1 expression and gender. The fixed-effects model was used to pool these researches as no statistical heterogeneity was observed between studies (*P*_Q−test_ > 0.1, *I*^2^ < 50%). The pooled results indicated that there was no significant relationship between NRP1 expression and gender (male versus female: OR = 0.776, 95%CI = 0.601–1.001, *P* = 0.051).

#### 3.2.2. Age

Nine studies [8–10, 16–21] reported the relationship between NRP1 expression and age (<60 versus ≥60). The fixed-effects model was used to pool these researches as no statistical heterogeneity was observed between studies. The pooled results indicated that there was no significant relationship between NRP1 expression and age (OR = 0.983, 95%CI = 0.750–1.288, *P* = 0.900).

#### 3.2.3. Stage

Three studies assessed [16, 18, 19] the relationship between NRP1 expression and stage. The fixed-effects model was used to pool these researches as no statistical heterogeneity was observed between studies. The pooled results indicated that there was a significant association between NRP1 expression and the tumor stage of gastric cancer (early stage versus advanced stage: OR = 0.128, 95%CI = 0.059 − 0.277, *P* ≤ 0.001).

#### 3.2.4. Tumor Size

**Figure 2:**
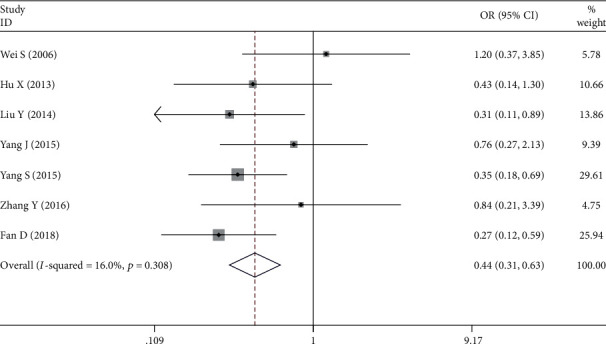
Forest plot about the association between neuropilin 1 expression and tumor size in gastric cancer (less than 5 cm versus more than 5 cm).

Seven studies assessed [8–10, 16–19] the relationship between NRP1 expression and tumor size. The fixed-effects model was used to pool these researches as no statistical heterogeneity was observed between studies (Figure 2). The pooled results indicated that there was a significant association between NRP1 expression and the tumor size of gastric cancer (tumor size less than 5 cm versus more than 5 cm: OR = 0.443, 95%CI = 0.310 − 0.632, *P* ≤ 0.001).

#### 3.2.5. Laurèn's Classification

Two studies [8, 21] assessed the relationship between NRP1 expression and Laurèn's classification. The fixed-effects model was used to pool the researches as no statistical heterogeneity was observed between studies. The results indicated that there was no significant relationship between NRP1 expression and Laurèn's classification (Lauren diffuse type versus intestinal type: OR = 0.729, 95%CI = 0.445–1.193, *P* = 0.208).

#### 3.2.6. pTNM Stages

**Figure 3:**
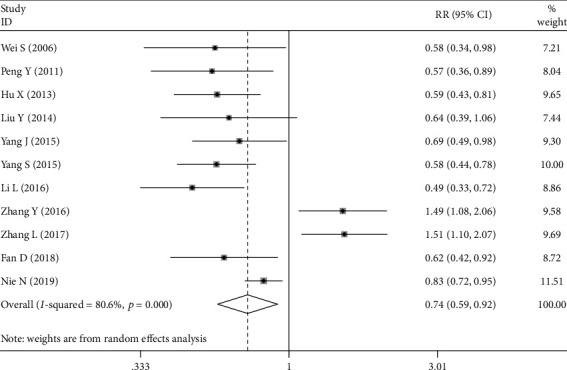
Forest plot about the association between neuropilin 1 expression and TNM stages in gastric cancer (stage I-II group versus stage III-IV group).

Eleven studies [7–10, 16–21, 23] assessed the relationship between NRP1 expression and pTNM stages. The random-effects model was used to pool these researches as statistical heterogeneity was observed between studies (*P*_Q−test_ < 0.1, *I*^2^ > 50%). As shown in Figure 3, NRP1 expression rate in patients with stages I and II gastric cancer was much lower than those with III and IV gastric cancer (OR = 0.736, 95%CI = 0.589 − 0.919, *P* = 0.007).

#### 3.2.7. Differentiation

**Figure 4:**
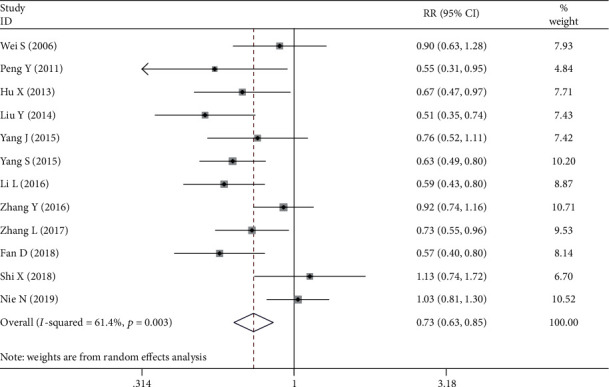
Forest plot about the association between neuropilin 1 expression and differentiation in gastric cancer (well to medium differentiation group versus poor differentiation group).

All included studies assessed the relationship between the NRP1 expression and histologic type. The random-effects model was used to pool these researches as statistical heterogeneity was observed between studies (Figure 4). The results indicated that well/moderate differentiation patients of gastric cancer had a much lower NRP1 expression rate versus poor differentiation group (OR = 0.735, 95%CI = 0.632 − 0.854, *P* ≤ 0.001).

#### 3.2.8. Metastasis of Lymph Node

**Figure 5:**
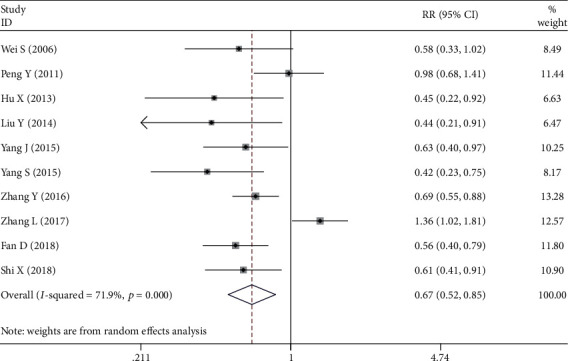
Forest plot about the association between neuropilin 1 expression and metastasis of lymph node in gastric cancer (non-lymph node metastasis group versus lymph node metastasis group).

Ten studies [7–10, 16–19, 21, 22] assessed the relationship between the NRP1 expression and metastasis of lymph node. The random-effects model was used to pool these researches as statistical heterogeneity was observed between studies. As shown in Figure 5, NRP1 expression rate in patients without lymph node metastasis was much lower than those with lymph node metastasis (OR = 0.667, 95%CI = 0.522 − 0.854, *P* = 0.001).

### 3.3. Publication Bias

**Figure 6:**
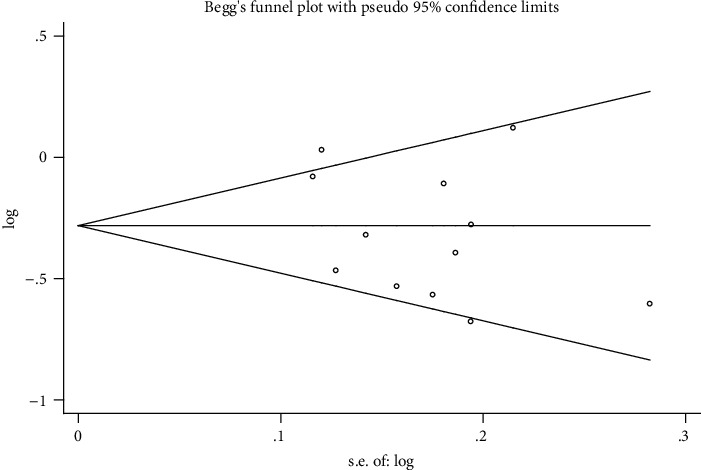
Begg's funnel plot (*P* = 0.493).

**Figure 7:**
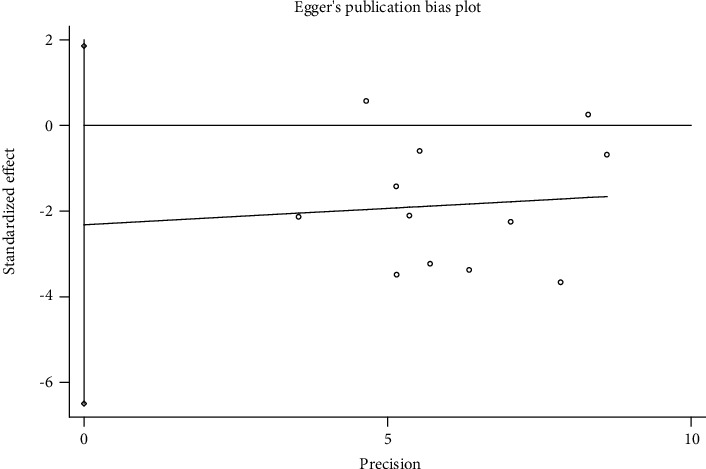
Egger's publication bias plot (*P* = 0.244).

Begg's funnel plot (Figure 6) and Egger's publication bias plot (Figure 7) did not show any evidence of obvious asymmetry. The statistical test indicated no potential publication bias (*P* > 0.05).

## 4. Discussion

NRP1 is involved in the development of cardiovascular system and the pathogenesis of cancer with an important role in angiogenesis. Studies indicated that NRP1 is abnormally expressed in a variety of tumor cells, including gastric cancer. NRP1 can participate in tumor development and promote tumor metastasis by eliciting a range of intracellular signaling cascades [24, 25]. Published data have shown that the analysis of NRP1 expression levels could provide a predictive marker of clinical outcome and prognosis in gastric cancer [5, 21, 26, 27]. Also, it is an exciting and challenging endeavor to employ NRP1-inhibitory strategies for cancer treatment [6, 28]. Therefore, studies on the relationship of NRP1 expression and clinicopathological characteristics of GC by IHC emerged with inconclusive results from different publications.

To systematically investigate the relationship between the expression of NRP1 protein and clinicopathological features of gastric cancer, the present study screened the published literature regarding the expression of NRP1 in gastric cancer by immunohistochemistry, and pooled the available data by meta-analysis. A total of 12 studies including 1225 gastric cancer patients were included in the analysis. The results showed that the positive rate of NRP1 protein expression in gastric cancer was higher in those with tumor larger than 5 cm *versus* with tumor smaller than 5 cm, higher in those with stages III-IV than with stages I-II, higher in low differentiation than well/moderate differentiation, and higher in those with lymph node metastasis than without lymph node metastasis. There was no statistical significance association between the expression of NRP1 protein and gender, age, clinical stage, and Laurèn's classification in gastric cancer.

The main disadvantages in our meta-analysis include (1) all the included studies are from China and ethnicity was not identified within each study; (2) the number of samples included in the study is limited; also, the focused of reports are various. For example, 7 studies provide relevant data for analysis about the comparison of tumor size, while only 2 items have relevant data about Laurèn's classification; (3) in terms of detection methods, although all of them are measured by immunohistochemistry, it is inevitable some differences among different research groups and different operators, such as the source of antibodies, specific experimental steps, dilution concentration, result judgment criteria, and other factors. The above may also be the main reason for the heterogeneity between studies.

## 5. Conclusions

In conclusion, the current studies show that NRP1 protein expression in gastric cancer is related to tumor size, TNM stage, differentiation degree, and lymph node metastasis and has a higher positive rate in patients of tumor size over 5 cm, TNM stages III-IV, low differentiation, and with lymph node metastasis. The detection of NRP1 protein expression might be useful to determine the lymph node metastasis in patients with gastric cancer. Further studies with larger samples and different ethnicities are required to confirm an association between NRP1 protein expression and the clinicopathological features of gastric cancer.

## Figures and Tables

**Table 1:** Characteristics and quality evaluation of included studies.

First author	Year	Language	Country	GC number	Gender (male/female)	NOS score
Wei S	2006	Chinese	China	60	43/17	8
Peng Y	2011	Chinese	China	63	42/21	7
Hu X	2013	Chinese	China	62	50/12	6
Liu Y	2014	Chinese	China	65	42/23	8
Yang J	2015	Chinese	China	72	57/15	7
Yang S	2015	Chinese	China	168	101/67	8
Li L	2016	English	China	141	87/54	7
Zhang Y	2016	Chinese	China	60	35/25	7
Zhang L	2017	English	China	203	133/70	7
Fan D	2018	Chinese	China	109	68/41	7
Shi X	2018	Chinese	China	94	54/40	7
Nie N	2019	Chinese	China	128	79/49	7

## Data Availability

The raw data supporting this meta-analysis are from previously reported studies, which have been cited. The processed data are included within the article. The full processed data in detail are also available from the corresponding author upon request.
